# Dietary Fibre for the Prevention of Post-Pancreatitis Diabetes Mellitus: A Review of the Literature and Future Research Directions

**DOI:** 10.3390/nu16030435

**Published:** 2024-02-01

**Authors:** Xinye Li, Maxim S. Petrov

**Affiliations:** School of Medicine, University of Auckland, Auckland 1023, New Zealand

**Keywords:** dietary fibre, nutrition, acute pancreatitis, post-pancreatitis diabetes mellitus

## Abstract

Post-pancreatitis diabetes mellitus—the most common sequela of pancreatitis—leads to poorer glycaemic control compared with type 2 diabetes. Because post-pancreatitis diabetes mellitus is an exemplar of secondary diabetes (with a clear underlying cause), much post-pancreatitis diabetes mellitus is preventable or treatable early. Earlier literature established the important role of dietary fibre in reducing plasma glucose in individuals with type 2 diabetes. The present review benchmarks available evidence on the role of habitual dietary fibre intake in pancreatitis and post-pancreatitis diabetes mellitus. It also paves the way for future research on the use of dietary fibre in the post-pancreatitis setting.

## 1. Physicochemical Properties of Dietary Fibre

The concept of dietary fibre was first introduced in the 1950s when it was observed that some aspects of the diet that are related to “fibre” reduce the incidence of toxaemia in pregnancy [1]. However, the definition of dietary fibre has been a subject of debate in the ensuing half-century, as it needs to encompass a wide range of dietary fibre types and related components. In 2010, the Codex Alimentarius Commission—an international food standards body established jointly by the World Health Organization and the Food and Agriculture Organization—published a definition of dietary fibre that met with broad international acceptance [2]. It characterises dietary fibre as carbohydrate polymers (that are not hydrolysed in the small intestine of humans) with at least 10 monomeric units [2]. Unlike many other nutrients, dietary fibre exerts its health effects mainly through its structural and chemical properties during digestion in the gastrointestinal tract.

### 1.1. Particle Size, Porosity, and Hydration Properties

Several physicochemical properties of dietary fibre (e.g., solubility, viscosity, fermentability) are closely linked to the role of dietary fibre in human physiology. The journey of dietary fibre in the human gastrointestinal tract involves several events during which its particle size changes. Before dietary fibre can reach the large intestine for fermentation, it is mechanically broken through chewing in the mouth and grinding in the stomach. In addition, food processing procedures (such as cooking and milling) result in alterations in the particle size and structure of dietary fibre, coupled with the mechanical breakdown in the mouth. This alters the digestibility of fibre and the degradation of other plant compounds [3]. For example, raw starch granules present in the plant cells can become soluble after cooking. Second, the level of porosity and available surface area of dietary fibre affect its ability to bind with other particles in the gut, such as enzymes and bacteria. It also affects the fermentability of dietary fibre by the gut microbiota. Third, modifications to the plant cell-wall structure through industrial processing (such as cooking, drying, heating, and pH alteration) affect the hydration properties of dietary fibre, including swelling capacity, water-binding capacity, and water absorption [4].

### 1.2. Solubility and Viscosity

The solubility of dietary fibre is closely related to its role in physiology. In general, fibre that is relatively more soluble in water/solution tends to have a more branched structure (energetically unstable), which means it is less stable in its solid state. However, there are exceptions. For example, β-glucan with β(1,4) linkages is insoluble, whereas β(1,3)(1,4) linkages make β-glucan soluble [5,6]. The concept of viscosity is linked with the solubility of dietary fibre. Viscosity is defined as resistance to flow and is linked to the capacity of dietary fibre to form a viscous solution in a concentration-dependent manner when dissolved [7]. Solubility is generally positively associated with viscosity. Therefore, soluble dietary fibre is more likely to form a gel-like substance and increase the viscosity of the gut’s contents compared with insoluble dietary fibre. The structure of soluble fibre determines how it forms a viscous solution. Soluble fibre with random-coil polysaccharides interacts with water molecules through entanglement, which then increases the viscosity. At the same time, soluble fibre with ordered-assembly polymers forms a gel network when divalent ions are present [8].

### 1.3. Interplay with Bile and Pancreatic Juice

Dietary fibre may interact with other chemical compounds in the gut, including bile acids and pancreatic enzymes. Primary bile acids are produced in the liver and secreted with bile, which is then converted into secondary bile acids upon bacterial fermentation. Bile acids are required for the digestion and absorption of lipids because they play a key role in the emulsification of lipids and the digestion and absorption of the lipophilic compounds [9]. When bile acids bind with dietary fibre in the gut, up to 95% of the bile acids (both primary and secondary bile acids) are no longer reabsorbed back through the enterohepatic circulation for the synthesis of bile acids. Instead, bile acids are excreted as bile salts in faeces [10]. The activity of pancreatic enzymes is known to be suppressed by several types of dietary fibre, including both soluble (e.g., pectin, guar gum) and insoluble (e.g., cellulose, wheat bran) ones. Pancreatic enzymes are secreted into the gut for the digestion and absorption of macronutrients and micronutrients. Dietary fibre alters the conformation of pancreatic enzymes and competes with nutrients for the binding site, which affects the catalytic activity performed by enzymes [11,12]. Pectin is known to suppress the activity of lipase [13,14]. Guar gum and cellulose are associated with the inactivation of α-amylase [15,16,17]. An in vitro study by Birkner and Kern found that adding dietary fibre (wheat bran, pectin, and guar gum) to human duodenal juice led to suppression of the activity of bile acids [18]. Similar findings were reported in other in vitro studies where slower nutrient digestion occurred when pancreatic enzymes were inhibited by both insoluble fibre (such as cellulose and rice bran) and soluble fibre (such as pectin) [17,19,20]. However, to date, no human in vivo study investigating the effects of dietary fibre on pancreatic enzymes has been conducted.

## 2. Physiological Effects of Dietary Fibre

### 2.1. Effects on Nutrients’ Availability

Most nutrients do not exist on their own and are often combined with other nutrients or compounds in the human diet. Specifically, dietary fibre rarely appears on its own in natural food and is often present in the form of a plant cell wall with other nutrients (such as starch, vitamins, and minerals) [21,22]. The primary plant cell wall is the most common form of dietary fibre in the human diet, with a structured network of cellulose and hemicellulose embedded in a network of pectin, while the secondary plant cell wall is the less common form, which is made up of lignin and cellulose [23]. The cell-wall structure affects the bioavailability of other nutrients (such as resistant starch 1) present in plant-based foods (such as vegetables and fruit). When the cell-wall structure remains intact, intracellular compounds (such as lipids and starch) are not available for absorption. During digestion, a series of events lead to structural changes in dietary fibre. This occurs through the intense mechanical breakdown of the plant cell wall, which allows the soluble components to dissolve or become hydrated. Evidence from studies of various plant-based foods (e.g., carrots, raw fruit, vegetables) showed thickened cell walls, reduced particle size, and solubilisation of pectin during digestion, which lead to a decrease in cell-wall integrity and an increase in cell-wall permeability [24,25]. As a result, nutrients within the plant cells become available for digestion. However, not all nutrients are absorbed before reaching the large intestine. Some of the nutrients may be released when dietary fibre is fermented by the gut microbiota, and these are still available for absorption through the colon’s epithelium (e.g., polyphenols). 

Dietary fibre can influence the bioavailability of micronutrients—both minerals and vitamins [26]. For example, dietary fibre (e.g., fructan) can enhance the absorption of calcium [27,28]. It is believed that an increase in dietary fibre intake results in a lower pH level in the gut, which increases the solubility of calcium ions [29,30]. The effects of dietary fibre on vitamins remain unclear due to mixed results from previous studies [31,32,33]. This gap in knowledge is of particular concern in the setting of chronic pancreatitis and its sequelae (such as new-onset diabetes mellitus) because of the accompanying malabsorption, which frequently leads to vitamin D deficiency. It is possible that, in individuals with exocrine pancreatic insufficiency and/or malnutrition, fibre acts as “anti-nutrients”—a concept derived from in vitro studies, suggesting that compounds present in plants may have untoward effects on certain biological systems. “Anti-nutrients” that act as pancreatic lipase inhibitors have been reviewed elsewhere [34].

### 2.2. Effect on Glucose Metabolism 

Another physiological effect of dietary fibre is its ability to reduce blood glucose levels. Most studies on the health effects of dietary fibre have focused on its effects on gastric emptying and nutrient absorption [35]. It is believed that the glucose-lowering effect is achieved directly through the gel-forming ability of soluble dietary fibre, which results in the gut’s contents becoming more viscous in the stomach and the intestine. In turn, increased viscosity of the gut’s contents in the stomach and intestine leads to slower nutrient diffusion, slower gastric emptying, and delayed nutrient absorption in the small intestine [36,37]. As a result, carbohydrates are absorbed into the circulation at a reduced rate, and with less fluctuation in postprandial glycaemia [38]. Insoluble fibre has distinct characteristics, and its effects on glucose metabolism are different from those of soluble fibre. It was initially thought that insoluble fibre may reduce the risk of diabetes through the production of short-chain fatty acids in the colon and their effects on hepatic insulin sensitivity [39]. More recent thinking is that high insoluble fibre intake may improve insulin resistance independent of weight loss, by interfering with the absorption of dietary protein [40]. This is an ongoing area of active research, and future high-quality studies are expected to conclusively establish the mechanism by which insoluble fibre affects blood glucose control.

Studies of healthy individuals have found that intake of dietary fibre is associated with improved glycaemic control. Food and colleagues conducted a randomised controlled trial of 11 healthy individuals, where the participants were given either fibre-enriched bread (flax fibre) or control bread (white bread) to eat after overnight fasting. The authors found that the intake of flax-fibre-enriched bread was associated with a notable reduction in peak postprandial glucose levels when compared with the control group [41]. Similar findings were reported in a crossover randomised controlled trial of 16 healthy men: the intake of breakfast with a high amount of insoluble fibre (33 g) was associated with a significant reduction in glycaemic response after 75 min, compared with the intake of breakfast with a low amount of insoluble fibre (1 g) [42]. A randomised controlled trial by Weickert and colleagues in overweight and obese women showed that the consumption of insoluble-fibre-enriched white bread (compared with regular white bread) for 3 days significantly increased whole-body insulin sensitivity in overweight and obese women [43]. Meta-analyses showed a significantly reduced risk of type 2 diabetes with higher fibre intake (in particular, cereal fibre intake) [44,45]. The role of dietary fibre in type 2 diabetes and prediabetes has been discussed comprehensively in previous studies and is beyond the scope of the present paper [46,47].

### 2.3. Effect on Lipid Metabolism

As far as the lipid-modulating effect of dietary fibre is concerned, evidence suggests that the interaction of dietary fibre with bile acids is the main contributor. A randomised controlled trial of a diet characterised by whole grains, legumes, and fruits and vegetables, compared with a diet high in refined grains, found that increased intake of dietary fibre leads to higher levels of circulating bile acids in healthy adults [48]. Since cholesterol is the precursor of bile acids, binding with dietary fibre results in increased excretion of bile acids in the form of bile salts [10,49]. As a result, the liver is required to synthesise bile acids using endogenous cholesterol, reducing serum cholesterol levels. Second, bile acids are essential for the emulsification of lipids and the digestion and absorption of lipophilic compounds. When dietary fibre is present, bile acids are no longer available for lipid emulsification. As a result, absorption of lipids and lipophilic compounds becomes reduced, which leads to reductions in serum triglycerides and total cholesterol [50]. In a 2023 large meta-analysis of randomised controlled trials, soluble fibre supplementation was shown to result in significant reductions in total cholesterol, LDL cholesterol, and apolipoprotein B [51]. The lipid-modulating effect of dietary fibre looks particularly promising in light of the PANDORA (PANcreatic Diseases Originating from intRa-pancreatic fAt) hypothesis, which postulates that excess fat in the pancreas is the main driver of all common diseases of both the endocrine (e.g., diabetes mellitus) and exocrine (e.g., pancreatitis) pancreas [52].

### 2.4. Effects on Gut Transit Time and Stool Mass

Dietary fibre is well known for its ability to influence colonic bulk (stool mass) and gut transit time. Dietary fibre, especially soluble fibre, can increase the water-binding activity in the colon and decrease the gut transit time. This is largely attributed to soluble fibre with ordered-assembly polymers (e.g., pectin), which allow the fibre to form gel networks in the colon when hydrated. A systematic review of healthy individuals showed that the gut transit time decreased with an increased intake of wheat fibre [53]. In addition, it was demonstrated that the bigger the particles of dietary fibre, the greater the stool mass when compared with refined dietary fibre particles [54]. Evidence suggests that both soluble and insoluble dietary fibre can influence stool mass. Soluble fibre increases the viscosity of the gut’s contents and makes them viscoelastic, which is positively associated with increases in stool bulk and mass [55,56]. By contrast, insoluble fibre stimulates mucus secretion and colonic muscular contraction, which contribute to stool mass and stool consistency [57,58,59].

### 2.5. Interplay with Gut Microbiota

Over one thousand different bacterial species reside in the human intestine, especially in the colon [60]. There is an intricate relationship between bacterial species, the host, and the diet. Growing evidence suggests that the gut microbiota is vital for normal digestion. Undigested dietary fibre reaches the large intestine and is fermented extensively by the gut microbiota as an essential energy source for the bacterial species [61]. The gut microbiota is known to maintain the gut barrier by directly fighting against pathogenic bacteria or acting through a secondary product such as short-chain fatty acids (SCFAs), which are produced when dietary fibre is fermented by the gut microbiota [62,63]. Butyrate, propionate, and acetate account for 90–95% of all SCFAs that are produced in the gut, and their intraluminal fraction is 15%, 25%, and 60%, respectively [64]. Evidence suggests that dietary fibre is associated with an increased growth of the beneficial bacterial genera, such as *Bifidobacterium* and *Lactobacillus*, which have a common role in improving host immunity and enhancing gut development [65,66,67]. 

Earlier studies investigated the use of dietary fibre in the form of supplementation with either prebiotics or synbiotics. Prebiotics are defined as selective types of dietary fibre (e.g., inulin, fructooligosaccharide, and galactooligosaccharide) that provide health benefits to the host by stimulating the growth of bacterial species that help to maintain a healthy gut environment [68]. By contrast, synbiotics refer to mixtures of selective types of dietary fibre and bacterial species that provide health benefits to the host [69]. Similarly, the use of prebiotic or synbiotic supplementation has been shown to stimulate the growth of favourable bacteria such as *Bifidobacterium* and *Lactobacillus* [70]. In addition, a meta-analysis of randomised controlled trials in overweight or obese individuals found that the use of synbiotic supplementation was associated with significantly reduced levels of fasting plasma insulin, while the use of prebiotics was associated with reductions in plasma cholesterol and triglycerides [71]. Similar findings were also observed in another meta-analysis of randomised controlled trials of individuals with type 2 diabetes [72].

One of the potential mechanisms by which prebiotics or synbiotics improve glucose and lipid parameters is through the production of SCFAs. High intake of dietary fibre, especially dietary fibre with high fermentability (e.g., β-glucan, pectin, fructooligosaccharide, inulin), is associated with increased production of SCFAs by the gut microbiota [73,74,75,76,77]. SCFAs exert an important role in the gastrointestinal tract. First, high levels of SCFAs in the large intestine help maintain the gut environment by inhibiting the growth of pathogenic bacterial species (such as *Salmonella* spp. and *Escherichia coli*) by lowering the pH level [78,79]. Second, SCFAs reduce the gut transit time together with dietary fibre through stimulating colonic contractile activity [80]. Third, SCFAs may have an immunomodulatory effect through regulating both the size and the function of the regulatory T-cell pool, which is beneficial for the mucosal immune system [81]. Fourth, SCFAs are associated with better gut barrier integrity through strengthening the tight junctions between intestinal epithelial cells [82]. Lastly, dietary fibre also exerts its glucose-lowering effect through the SCFA pathway through metabolic activities in the liver, including decreasing gluconeogenesis [83,84]. While the above evidence is mainly related to soluble fibre, it is worth noting that insoluble fibre is also involved in the interplay with the gut microbiome [85]. First, insoluble fibre provides a substrate that supports the diversity of the microbiome (generally associated with favourable health outcomes). Second, the fermentation of insoluble fibre can contribute to immune modulation. Third, the fermentation products of insoluble fibre (e.g., butyrate) contribute to the maintenance of the gut barrier. Butyrate serves as the primary energy source for colonocytes and has anti-inflammatory properties [86,87]. It is worth noting that different types of insoluble fibre may have varying effects on the gut microbiome, and the overall impact depends on factors such as the existing composition of the gut microbiome, the individual’s habitual diet, and the specific fibre consumed [88].

### 2.6. Interplay with Gut Hormones

Upon ingestion of food, the gastrointestinal tract secrets several different hormones, such as the incretin hormones and oxyntomodulin [89,90]. These gut hormones are secreted to regulate satiety, carbohydrate absorption, gastric emptying, and glucose metabolism [91,92]. Incretin hormones, such as glucose-dependent insulinotropic polypeptide (GIP) and glucagon-like peptide 1 (GLP-1), are responsible for augmenting the insulin-secretory response initiated by hyperglycaemia [93]. GIP was initially recognised as a gut hormone that inhibits gastric acid secretion (GIP was previously known as gastric-inhibitory polypeptide), but it was found that the main action of GIP is to stimulate insulin secretion [94]. GIP is produced from the enteroendocrine K cells located in the mucosa of the duodenum and upper jejunum, while GLP-1 is produced from the intestinal L cells located throughout the ileum and colon [95]. When nutrients, especially starch and sucrose, reach the gut, where the enteroendocrine K and intestinal L cells are located, GIP and GLP-1 are secreted and bind to their receptors in the cell membranes of pancreatic β-cells, which leads to an increase in insulin secretion [93,96]. The actions of incretin hormones go beyond glucose homeostasis. For example, evidence has suggested their roles in promoting weight loss [97,98], enhancing lipoprotein lipase activity and promoting fat storage in subcutaneous adipose tissue [99,100], delayed gastric emptying (GLP-1 only) [101,102], delayed absorption of nutrients in the intestine [103], slower intestinal transit [104], and limiting bone resorption [105]. 

Dietary fibre intake has been associated with an early response of incretin hormones. A randomised controlled trial by Weickert and colleagues demonstrated that intake of cereal-fibre-enriched bread (matched portion) by healthy individuals resulted in earlier insulin responses and reduced postprandial glucose responses [106]. The study also found that an increase in dietary fibre intake was associated with an earlier response of GIP [106]. Similarly, a crossover randomised controlled trial by Ames and colleagues found that healthy individuals who consumed tortillas with high soluble β-glucan contents (11.6 g) for at least 1 week had a lower glycaemic response (no significant increase in postprandial blood glucose and less fluctuation in postprandial plasma insulin levels) compared with the group that consumed glucose drinks instead [107]. Furthermore, this study found that the other intervention group, which consumed high-insoluble-fibre tortillas (19.6 g), had an earlier GLP-1 response and higher plasma levels of GLP-1 [107]. These findings suggest that the intake of dietary fibre may exert a glucose-lowering effect through the GIP/GLP-1 pathway (along with other mechanisms).

Oxyntomodulin, another gut hormone, is secreted from the intestinal L cells along with GLP-1. It was first identified and named after its ability to regulate gastric acid secretion, and it was later proven to be involved in glucose metabolism through binding and activating GLP-1 and glucagon receptors [108,109]. Other actions of oxyntomodulin have also been proposed [110]. For example, a study in individuals with obesity showed that oxyntomodulin could lead to weight reduction through suppressing appetite and increasing energy expenditure [111]. While many studies have investigated the relationship between dietary fibre and incretin hormone activities, there is a paucity of studies investigating the relationship between oxyntomodulin and dietary fibre intake. However, a 2021 randomised controlled trial by Di Mauro and colleagues demonstrated that oxyntomodulin may be influenced by dietary fibre intake [112]. Findings from this study showed that, among obese/overweight individuals with type 2 diabetes, both a Mediterranean diet (4.8 g of fibre) and a high-fibre vegetarian diet (23 g of fibre) elicited significant increases in postprandial oxyntomodulin levels [112]. Therefore, it is possible that an increase in dietary fibre intake could stimulate the release of oxyntomodulin postprandially, which may, in turn, contribute to better glycaemic control. More detailed information on the impact of soluble fibre on incretins and other gastrointestinal hormones was comprehensively presented in a recent review [113].

## 3. Pancreatitis and Post-Pancreatitis Diabetes Mellitus

The pancreas is a dual-functional organ composed of the exocrine and endocrine glands. This gives rise to the key functions of the pancreas, which are the digestion of nutrients through secreting pancreatic enzymes and regulating glucose homeostasis through the actions of pancreatic hormones. Therefore, it is rather intuitive that both the endocrine and exocrine parts of the organ are reciprocally affected by pathological processes in the pancreas [114]. Pancreatic cancer, as the most ominous pancreatic disease, is one of the leading causes of cancer-specific mortality, with a dismal 5-year survival rate [115]. It has been shown that both acute pancreatitis (AP) and chronic pancreatitis (CP) are risk factors for developing pancreatic cancer. Studies have found that individuals with CP are at a 7.9-fold higher risk of developing pancreatic cancer within 5 years of the initial diagnosis of CP [116]. Regarding AP, studies from Denmark and Sweden have demonstrated that individuals with AP are at higher risk of pancreatic cancer [117,118]. A study from Denmark found that, compared with the general population, individuals with AP are at a 2-fold higher risk of developing pancreatic cancer after matching by age and sex [119]. Similarly, a study from Sweden found that individuals with AP have a 2.2-fold higher risk of pancreatic cancer after adjustment for the recurrence of AP and CP after follow-up for 5 to 10 years [118].

Diabetes of the exocrine pancreas (DEP) is a common type of secondary diabetes [114,119,120]. DEP includes several nosologies, including post-pancreatitis diabetes mellitus (PPDM), pancreatic-cancer-related diabetes, and cystic-fibrosis-related diabetes [121]. PPDM is the most frequent sequela of AP and the most common subtype of DEP [119]. Since AP and CP are the two main types of pancreatitis, PPDM also has two subtypes: post-acute-pancreatitis diabetes mellitus (PPDM-A, the largest contributor to PPDM) and post-chronic-pancreatitis diabetes mellitus (PPDM-C) [120]. In the past, it was thought that the development of diabetes after pancreatitis only occurs after CP, or when pancreatitis results in pancreatic necrosis, which leads to a loss of pancreatic β-cell function [119]. However, it has recently been hypothesised that the natural course of PPDM represents a continuum, which is characterised by a gradual progression from insulin resistance after the first episode of non-necrotising AP to the loss of pancreatic β-cell function due to low-grade inflammation accompanied by end-stage CP [114,119]. It is important to acknowledge that most of the evidence to date has come from cross-sectional studies and, therefore, causality cannot be inferred. In particular, it is possible that individuals with pre-existing non-diabetic insulin resistance have a higher chance of acquiring AP (and maintaining insulin resistance when acquiring PPDM-A). While it remains to be established conclusively that sustained low-grade inflammation after AP induces novel insulin resistance, recent findings from the longitudinal LACERTA cohort study do indicate that fasting insulin is a significant risk factor for new-onset derangements in glucose metabolism following an attack of AP [122]. Other key factors involved in the pathogenesis of PPDM include intra-pancreatic fat deposition, altered lipid metabolism, and dysfunction of the pancreas–gut–brain axis [49,123,124]. Detailed recommendations on the differential diagnosis of PPDM and type 2 diabetes have been published elsewhere [115].

Several high-quality studies have established that the risk of developing PPDM is associated with a history of AP [125,126,127,128]. The 2020 prospective longitudinal LACERTA study by Bharmal and colleagues showed that individuals are at a higher risk of developing PPDM after an episode of AP [125]. The authors found that 40% of individuals developed new-onset prediabetes or diabetes within two years of an AP attack [125]. When looking at PPDM-A, a 2014 meta-analysis of 24 prospective clinical studies found that 23% (95% CI: 16 to 31%) of individuals developed PPDM-A after an attack of AP (after excluding individuals with CP, prior history of diabetes or prediabetes, and pancreatic resection) [126]. Similarly, two studies conducted in Taiwanese adults after an attack of AP who were previously normoglycaemic showed that the adjusted risks of new-onset diabetes after pancreatitis were 2.15 and 2.54, respectively, when compared with the general population with no history of diabetes or AP [127,128]. In comparison with individuals with type 2 diabetes, individuals with PPDM have poorer glycaemic control. A cohort study from the UK confirmed that individuals who developed PPDM-A had significantly higher HbA1c levels when compared with individuals with type 2 diabetes at the time of diagnosis (67 mmol/mol vs. 63 mmol/mol, *p* = 0.002), at 1 year (54 mmol/mol vs. 51 mmol/mol, *p* < 0.001), and at 5 years (60 mmol/mol vs. 55 mmol/mol, *p* < 0.001) [129]. The authors also found that, when compared with individuals with type 2 diabetes, individuals with PPDM-A were 6.4 times more likely to use insulin therapy 1 year after the diabetes diagnosis, and 5.2 times more likely 5 years after the diabetes diagnosis, after adjustment for common covariates (i.e., age, sex, ethnicity, alcohol consumption, smoking status, body mass index, and initial haemoglobin level at diagnosis) [129]. The above findings suggest that, even when individuals with PPDM-A started insulin therapy earlier, it did not translate into better glycaemic control when compared with individuals with type 2 diabetes.

The long-term use of metformin has been associated with a notable reduction in the risk of mortality (adjusted HR: 0.5; 95% CI: 0.36 to 0.70) for individuals with PPDM-A [130]. Notably, this beneficial effect of metformin was still 25% more pronounced when compared with individuals with type 2 diabetes (adjusted HR: 0.75; 95% CI: 0.72 to 0.77) [130]. However, long-term use of insulin therapy in PPDM was not as promising as metformin in reducing the mortality rate. Specifically, when compared with individuals with PPDM who had never used insulin, the long-term use of insulin was associated with an increased risk of progression from the first episode of AP to recurrent AP or CP among individuals with PPDM who were insulin-naïve (adjusted HR: 1.56; 95% CI: 1.15 to 2.11), after adjustment for pancreatic-related factors (i.e., aetiology, severity, and time since last AP attack) [131].

When looking at PPDM in the long term, individuals who developed PPDM had a higher risk of health complications when compared with individuals with type 2 diabetes [114,132]. First, individuals with PPDM (80.5 per 1000 person-years) showed a higher all-cause mortality rate, by an excess of 14.8 deaths per 1000 person-years, when compared with individuals with type 2 diabetes (65.5 per 1000 person-years), which was mostly attributed to cardiovascular mortality (mortality rate: 25.2 per 1000 person-years) [132]. Second, PPDM results in a significantly higher cancer mortality rate (excluding pancreatic cancer) than type 2 diabetes [132]. When looking at pancreatic cancer separately, a 2020 population-based study found that individuals with PPDM are at a 6.9-fold higher risk (adjusted HR: 6.94; 95% CI: 4.09 to 11.77) of developing primary pancreatic cancer than individuals with type 2 diabetes alone [133]. Moreover, the study found that individuals with PPDM showed a 2.3-fold higher risk of pancreatic cancer when compared with individuals with diabetes before an attack of pancreatitis, after adjustment for covariates [133]. This finding suggests that the increased risk of pancreatic cancer in individuals with PPDM is not merely due to the impact of pancreatitis as a comorbidity in individuals with type 2 diabetes, but rather that pancreatitis applies an impact beyond being a comorbidity in individuals with PPDM [119]. Overall, to improve the health outcomes of this sequela of AP, it is crucial to intervene at an early stage, with a view to preventing PPDM.

## 4. Dietary Fibre in Pancreatitis and Post-Pancreatitis Diabetes Mellitus 

Studies of individuals with AP have shown that a high-fibre diet is associated with reduced length of hospital stay and decreased risk of complications [134,135]. Although investigations of the effects of dietary fibre in individuals with AP are scarce, evidence from the available studies suggests the potential effect of dietary fibre in improving health outcomes in individuals with AP. In 2007, Karakan and colleagues conducted a double-blind randomised controlled trial with 30 individuals with severe AP [135]. The intervention group that received fibre-enriched enteral nutrition (a total of 1.5 g/100 mL multi-fibre supplement as a prebiotic, including 0.7 g/100 mL soluble fibre and 0.8 g/100 mL insoluble fibre) showed significant reductions in hospital stays and overall complication rate compared with the control group who used the standard enteral nutrition formula. Another randomised controlled trial of 49 individuals with severe AP using fibre-enriched enteral feeding showed similar results [136]. The study found that the intervention group that received an extra 20 g of soluble fibre (polydextrose) in the enteral nutrition formula had significant reductions in feeding intolerance and plasma blood glucose when compared with the control group [136]. This reduction in blood glucose might be related to the improved intestinal gut function, absorption, and the direct glucose-lowering effect of soluble fibre [137]. Other notable studies that employed enteral feeding formulae were the two randomised controlled trials by Olah and colleagues [134,138]. The findings from these two trials showed significant reductions in pancreatic necrosis, organ failure, and systemic inflammatory response syndrome in the intervention groups (which received a fibre-enriched enteral tube feed and 10 g of oat fibre in the 2002 trial, and a 10 g mixture of four bioactive plant fibres—β-glucan, inulin, pectin, and resistant starch—in the 2007 trial). However, these two studies also used probiotics (along with fibre supplementation), making it impossible to determine whether it was dietary fibre that contributed to the improved clinical outcomes [139].

Clinical observations suggest that the use of dietary fibre in individuals with CP is associated with steatorrhoea, bloating, and abdominal distention [140]. It is believed that this is due to the maldigestion and malabsorption of fat in the gut, but also because dietary fibre suppresses pancreatic enzymatic activity [141]. A rodent study showed that 20% wheat bran supplementation for 2 weeks resulted in significantly elevated levels of pancreatic enzymes (i.e., lipase, amylase, and trypsin) [142]. Unfortunately, to date, there have been no human trials to investigate the effects of dietary fibre on pancreatic enzyme activity in individuals with CP.

Nutritional management of PPDM has only recently started to attract research attention [143]. The first study of habitual dietary fibre intake in individuals after an episode of pancreatitis was conducted by Li and colleagues in 2021 as part of the ANDROMEDA project [144]. This was a cross-sectional study of 108 individuals following an episode of acute pancreatitis. Habitual dietary fibre intake was determined using the EPIC-Norfolk food frequency questionnaire. Multivariable regression analyses were conducted, adjusting for covariates such as age, sex, BMI, energy intake, use of antidiabetic medications, aetiology of acute pancreatitis, recurrence of acute pancreatitis, and the presence of pancreatic necrosis. The study showed that increased habitual intake of dietary fibre was significantly inversely associated with fasting plasma glucose in individuals with PPDM-A. This held true for total fibre, soluble fibre, and insoluble fibre. In particular, every 1% increase in the intake of total fibre, soluble fibre, and insoluble fibre was associated with a 0.15%, 0.13%, and 0.13% decrease in fasting plasma glucose, respectively. In the analysis of common sources of dietary fibre, the authors showed that increased intake of vegetables and nuts (but not of fruit and cereals) was significantly inversely associated with a reduction in fasting plasma glucose [144].

## 5. Directions for Further Research

Based on the findings presented above, several aspects require further investigations through purposely designed studies in individuals after an attack of AP. First, longitudinal studies and randomised controlled trials are warranted to investigate the causal relationships between dietary fibre and markers of glucose metabolism in the post-pancreatitis setting. Similar to studies in individuals with type 2 diabetes [106,145], randomised controlled trials may consider administering isocaloric bread or liquid with different amounts of dietary fibre to individuals with PPDM-A. In addition, future randomised controlled trials could look at introducing an equal number of different types of fibre (such as pectin versus β-glucan) in isocaloric meals or food to elucidate the effectiveness of each type of fibre in individuals after AP. Also, longitudinal studies could look at the relationship between dietary fibre and postprandial (as opposed to fasting) plasma glucose levels, for more comprehensive investigation in the context of post-pancreatitis glucose derangement.

Second, the use of prebiotics and synbiotics in individuals with metabolic diseases has been shown to be effective in improving hyperglycaemia and dyslipidaemia [146]. However, there are controversies around the dose of prebiotics (e.g., specific dietary fibres such as inulin, fructooligosaccharide, and galactooligosaccharide) when used in combination with bacterial species in synbiotic products. Many trials administered less than 1 g of dietary fibre as the prebiotic component, which is not in line with longitudinal and randomised controlled studies that investigated the sole use of dietary fibre in different disease settings [147,148,149]. Little is known about the effects of dietary fibre when used in combination with probiotics. Therefore, the effects of dietary fibre should be established by future randomised, double-blind, placebo-controlled trials to confirm the independent role of dietary fibre in regulating glucose homeostasis and other relevant parameters (e.g., intra-pancreatic fat deposition and lipid metabolism) in the post-pancreatitis setting. Future studies could also introduce synbiotics with different doses of dietary fibre to elucidate whether there are dose-dependent effects of synbiotics on the gut microbiota profile and glucose homeostasis.

Third, studies have suggested that dietary fibre is associated with the production of SCFAs, the alteration of the gut microbiota profile, the levels of circulating bile acids, and the earlier stimulation of incretins. However, the potential links between them and the underlying pathophysiology of how these mechanisms result in reductions in plasma glucose are still poorly understood in the post-pancreatitis setting. Future randomised controlled trials are warranted to investigate the underlying pathophysiological events in post-pancreatitis individuals. For example, randomised controlled trials could administer different amounts of dietary fibre to individuals with PPDM-A and compare the circulating levels of bile acids and incretins in association with changes in fasting plasma glucose and insulin traits. In addition, future longitudinal studies could look at the effects of different levels of habitual dietary fibre intake on the gut microbiota profile (i.e., bacterial species and levels of luminal and circulating SCFAs).

Fourth, numerous studies have demonstrated that PPDM is different from type 2 diabetes in terms of pathology, glycaemic control, and risk of other complications [114,119,120]. It is conceivable that nutrition therapy may have different effectiveness in individuals with different types of diabetes [150,151,152]. Although previous studies showed that nutritional interventions had little effect in individuals with a longstanding history of type 2 diabetes [153,154,155], no study to date has investigated this in individuals with PPDM-A. Purposely designed interventional studies are now warranted to compare the effects of dietary fibre intake between these two types of diabetes. For example, randomised controlled trials could recruit individuals with newly diagnosed type 2 diabetes, PPDM-A, diagnosed with type 2 diabetes > 5 years, and diagnosed with PPDM > 5 years, and administer an equal amount of dietary fibre in their meals to investigate the effects on glucose markers and insulin traits.

Last, recent studies have shown that there could be potential interplay between dietary fibre and other nutrients or compounds (e.g., dietary iron, resistant starch, and β-hydroxybutyrate), which may lead to indirect metabolic pathways involved in glucose homeostasis. Indeed, earlier studies demonstrated that dietary fibre could interfere with mineral and metal ions in the gut through its porous surface and affect nutrient availability. Therefore, to comprehensively investigate the effect of dietary fibre in regulating plasma glucose, future studies could introduce nutrients that are known to interplay with dietary fibre to determine the independent effect of dietary fibre on glucose homeostasis in individuals after pancreatitis. Future randomised controlled trials in individuals with PPDM could compare the markers of glucose metabolism between individuals with or without exocrine pancreatic dysfunction when they have the same amount of dietary fibre in each meal.

## 6. Conclusions

The present review brings to the fore the role of fibre supplementation in pancreatitis and post-pancreatitis settings. The use of fibre-enriched enteral formulae helps preserving the gut barrier and, hence, patients with acute pancreatitis may potentially benefit from fibre supplementation alone (i.e., without concurrent use of probiotics) during hospitalisation. After hospital discharge, increased intake of dietary fibre (specifically, vegetables and nuts) may benefit individuals after an attack of acute pancreatitis, with a view to preventing PPDM. Individuals with advanced chronic pancreatitis, exocrine pancreatic insufficiency, and/or malnutrition may not benefit from fibre supplementation, because of exacerbated malabsorption and steatorrhoea following the use of fibre. As the above inferences are based on a rather limited body of knowledge about individuals with diseases of the pancreas, high-quality clinical research on the use of dietary fibre in pancreatitis and its sequelae is warranted.

## Data Availability

Not applicable.
